# Multidisciplinary tumor boards in oral cavity cancer: survival effect due to balancing guideline adherence and treatment delays

**DOI:** 10.3389/froh.2024.1493319

**Published:** 2024-11-15

**Authors:** Valentin Burkhardt, Katharina El-Shabrawi, Sarah Riemann, Pit Voss, Christoph Becker

**Affiliations:** ^1^ Department of Otorhinolaryngology-Head and Neck Surgery, Medical Center—University of Freiburg, Faculty of Medicine, University of Freiburg, Freiburg, Germany; ^2^ Department of Oral and Craniomaxillofacial Surgery, Medical Center—University of Freiburg, Faculty of Medicine, University of Freiburg, Freiburg, Germany

**Keywords:** oral cavity cancer, multidisciplinary tumor board, survival analaysis, time to treatment, head and necek squamous cell carcinoma, head and neck cancer

## Abstract

**Objectives:**

The purpose of the study was to assess the impact of a pretherapeutic Multidisciplinary Tumor Board (MTB) presentation on the prognosis and treatment outcomes in patients with primary oral cavity carcinoma.

**Materials and methods:**

This single-center study included 630 patients diagnosed with oral cavity carcinoma treated between 2010 and 2020. The study cohort was divided in a group with and without pretherapeutic MTB presentation. Data on patient demographics, tumor characteristics, treatment and the time to treatment initiation (TTI) were collected retrospectively.

**Results:**

Primary findings revealed no significant difference in 3-year survival rate (3-YSR) and 3-year disease-free survival rate (3-YDFSR) for the non-MTB and MTB group. The 3-YSR was 73.1% in the non-MTB group and 67.1% in the MTB group (*p* *=* 0.112). The 3-YDFSR was 73.8% in the non-MTB group and 76.5% in the MTB group (*p* = 0.447). Estimated mean 5-year survival (5-YS) and 5-year disease-free survival in (5-YDFS) did not differ significantly between both groups, across the UICC stages I-IV, as well as for the entire cohort. The TTI was significantly longer in the MTB group (33.5 days, CI: 31.3;35.7) compared to the non-MTB group (20.1 days, CI: 17.9;22.4, *p* < 0.001). The MTB group adhered more frequently to the national guidelines (68% vs. 79.6%, *p* < 0.01).

**Conclusion:**

The results demonstrate both positive and negative side effects of the MTB presentation in patients with oral cavity cancer. Further multicenter studies will be required to assess the impact of TTI and adherence to guidelines on the survival of oral cavity cancer patients.

## 1 Introduction

In 2020, a total of 377,713 new cases of oral cancer were diagnosed worldwide, making it the eighth most common cancer in men and the fifteenth most common in women (1). In Germany, oral cavity and pharyngeal cancer account for 3.2% of newly diagnosed tumor cases in men and 1.4% in women (2).

Oral cavity cancer occurs in different subsites, including the anterior tongue, buccal mucosa, hard palate, lips, floor of the mouth, alveolar ridges, and retromolar trigone; the tongue is the most common subsite. Over 90% of oral cavity cancers are classified as squamous cell carcinomas, other entities such as adenocarcinomas are relatively rare (3, 4).

The German guideline was initially published in 2012 and recommends a primarily surgical approach comprised of a tumor resection and neck dissection, followed by adjuvant radio(chemo)therapy if indicated by factors such as close margin resection (<3–5 mm), perineural invasion, lymphovascular invasion, or positive lymph nodes on histopathological analysis (5). These recommendations are consistent with those set forth by the National Comprehensive Cancer Network (NCCN) (6). Reviews of multiple databases have demonstrated that patients with oral cavity cancer who underwent surgical treatment had a superior overall survival (OS) rate compared to those who did not receive surgical intervention (7, 8).

Whether cancer therapy should be delivered in a center-based fashion involving a multidisciplinary team (MDT) is unclear. A recent study conducted in Germany with over 497,000 patients with various cancer types demonstrated that patients treated in certified cancer centers exhibited improved OS (9). Certified centers encompass a number of factors that contribute to improved outcomes, including MDTs, highly experienced surgeons, superior nursing care, and a multidisciplinary tumor board (MTB).

The MTBs comprise a range of disciplines, the specific composition of which varies between clinics and countries. The MTB for head and neck cancer incorporates the primary treating disciplines, namely head and neck surgeons, otorhinolaryngologists, radiation oncologists, plastic surgeons and medical oncologists. Furthermore, they are responsible for the management of therapy and aftercare. In addition to the aforementioned disciplines, diagnostic radiologists and pathologists play a pivotal role in the MTB, contributing to the classification of histopathological sections and staging and providing expert advice to the primary treating disciplines (10).

The MTB is a widely adopted tool in the management and treatment of various cancer types and is typically integrated into large cancer centers (11). Consequently, studies must distinguish between the influences of the MDT and MTB (12). Several studies have reported higher treatment rates, faster initiation of treatment post-diagnosis, adherence to clinical guidelines, and increased OS caused by the MTB (11, 13–15). However, there have also been multiple studies that have been unable to demonstrate a survival benefit of MTBs for patients (16–18). A systematic review by Pillay et al. in 2016, encompassing various tumor types, confirmed limited evidence for a positive correlation between MTBs and improved OS of the patients (11).

There are few studies for MTB in treatment of head and neck cancer, often including a heterogenous group of patients, considering different regions within the head and neck (14, 19). Comparing cancers various head and neck regions may bias OS outcomes due to varying prognoses.

Despite the cost and labor-intensive nature of MTB, it is widely accepted and implemented in cancer centers worldwide. While there is limited evidence supporting its positive impact on patient outcomes, MTB plays a pivotal role in center-based cancer treatment. This study aims to evaluate the potential benefits of a pretherapeutic MTB for oral cavity cancer.

## 2 Material and methods

### 2.1 Retrospective analysis

This retrospective analysis evaluated data from all patients with oral cavity carcinomas treated at the University Medical Center Freiburg. This study was approved by the ethics committee (Local Ethics Committee Number: 312/20, approved 23 June 2020, registered DRKS00023378). The study included all patients diagnosed with cancer of the oral cavity between January first, 2010, and December 31, 2020. Patients were excluded if their initial diagnosis or treatment occurred elsewhere, if they had secondary or recurrent oral cavity carcinoma. Patients were included in the study if their cancers originated from the sites listed in the 2024 National Comprehensive Cancer Network (NCCN) guideline for oral cavity cancers (6).

The University Medical Center Freiburg convenes MTBs for head and neck cancer on a weekly basis and is comprised of physicians from various specialties, including otorhinolaryngology, craniomaxillofacial surgery, radiology, pathology, radiation therapy, and oncology. The patient's inclusion in the MTB is initiated by the physician of the department responsible for the patient's care.

A comparative analysis was conducted to examine patient demographics, cancer type and location, clinical and pathological staging, the duration from diagnosis to first MTB presentation, treatment initiation, treatment regimen, outcomes, follow-up periods, and death occurrences. Age at initial diagnosis, gender, carcinoma type, recurrences, secondary carcinomas, and therapy regimen were extracted from the patient records at the University Medical Center Freiburg. The date of diagnosis was defined as the date on which the pathological report was made available to the treating physicians. The commencement of treatment was defined as the date of the initial curative surgical procedure or the initiation of radiotherapy or chemotherapy. The interval between these two events was designated as TTI. The dates of follow-up and death for all cases were obtained from the national cancer register in November 2022. The duration of follow-up and time of death were measured in months from the date of initial diagnosis for determination of OS (20). The recurrence date was determined analogously to the date of initial diagnosis, utilizing pathological reports.

Patients were assigned retrospectively to the MTB group, if they were presented in the MTB prior to the initiation of treatment and to the non-MTB group if they weren't. All MTB-recommendations were noted using the MTB protocols. The non-MTB and MTB groups were partly predetermined by the implementation of an MTB at the University Medical Center Freiburg in 2014.

Tumor stages were adjusted to the 7th TNM classification from 2010 to ensure comparability for patients treated prior to the introduction of the 8th TNM classification in April 2017. Clinical TNM stages were determined using documents from the University Medical Center Freiburg, including MTB protocols, surgical reports, and doctor's letters. Pathological TNM stages were determined by assessing pathological reports and adjusting them to the pTNM classification of the 7th TNM classification after the introduction of the 8th TNM classification. This adjustment considered changes in TNM classification due to factors such as infiltration depth and extracapsular growth in lymph nodes.

### 2.2 Statistical analysis

Given the explorative character of the study and the differing subsets involved, adjustments for multiple testing were not applied. Prior to conducting tests, all variables were assessed for normal distribution using the Shapiro-Wilk test. The statistical methods used for analysis comparing distributions between the MTB and non-MTB group included the *t*-test, Shapiro-Wilk test and ANOVA-Regression for mean comparisons. In order to facilitate a comparison of the adherence to national guidelines, the chi-square test was employed. A comparison of TTI was conducted utilizing both *t*-test and ANOVA. OS was defined as the period between primary surgery and last contact with the patient, with a 5-year survival (5-YS) adjustment. 5-year disease-free survival (5-YDFS) was defined as the period between primary surgery and the detection of a recurrence within 5 years. 3-year survival rate (3-YSR) and 3-year disease-free survival rate (3-YDFSR) were calculated using the recurrences and deaths of patients within three years after primary diagnosis. Chi-square testing was employed to facilitate a comparative analysis between the 3-YSR and the 3-YDFSR.

Survival analysis included Kaplan-Meier curves, log-rank tests, and Cox proportional hazards regression. The hazard ratio (HR) with associated 95% confidence intervals (CI) and *p*-values were estimated for survival analyses. A statistically significant threshold was set at a *p-*value <0.05. Data analysis was performed using IBM SPSS Statistics 29 (IBM, Armonk, New York, USA).

## 3 Results

### 3.1 Cohort analysis

A total of 791 patients, which were treated at the University Medical Center Freiburg between 2010 and 2020, were screened. Of these, 161 patients were excluded due to data insufficiency (*n* = 80), oral cavity carcinoma as a secondary carcinoma (*n* = 62) or experiencing a recurrence of a previously treated oral cavity carcinoma (*n* = 19). The final cohort included 630 patients, of whom 238 patients received no pretherapeutic presentation in MTB and 392 patients received a pretherapeutic MTB presentation.

In the non-MTB group, there were 93 (39.1%) female patients and 145 (60.9%) male patients, whereas the MTB group consisted of 172 (43.9%) female patients and 220 (56.1%) male patients (Table 1). The mean age at the time of the initial diagnosis was 63.19 years in the non-MTB group and 65.34 years in the MTB group. Squamous cell carcinoma (SCC) was the most prevalent entity in both groups, with 219 (96.2%) cases in the non-MTB group and 383 (97.7%) cases in the MTB group. A total of 72 (30.3%) patients in the non-MTB group and 102 (26%) patients in the MTB group experienced recurrence during observation period. Secondary carcinomas were observed in 30 (12.6%) patients in the non-MTB group and in 46 (11.7%) patients in the MTB group. There were no statistically significant differences between the groups regarding gender distribution, age at initial diagnosis, recurrences, secondary carcinomas, and the distribution of cancer entities (all *p* *>* 0.05, Table 1).

**Table 1:** Cohort analysis data. The cohort was divided into two groups with and without a pretherapeutic MTB presentation.

	MTB	Non-MTB	*p* value
*N*	392	238	
Age at initial diagnosis (years)			0.057
Mean ± SD (median)	65 ± 14 (65)	63 ± 14 (63)	
Gender, *n* (%)			0.237
Male	220 (56.1)	145 (60.9)	
Female	172 (43.9)	93 (39.1)	
Entity, *n* (%)			0.112
Squamous cell carcinoma	370 (94.4)	212 (89.1)	
Adenocarcinoma	6 (1.5)	2 (0.8)	
Mucoepidermoid carcinoma	2 (0.5)	2 (0.8)	
Verrucous carcinoma	13 (3.3)	17 (7.1)	
Primary tumor—T, *n* (%)			<0.001
T 1	138 (35.2)	137 (57.6)	
T 2	116 (29.6)	63 (26.5)	
T 3	52 (13.3)	15 (6.3)	
T 4	85 (21.7)	23 (9.7)	
Regional lymph nodes—*N*, *n* (%)			<0.001
N 0	233 (59.4)	173 (72.7)	
N 1	52 (13.3)	34 (14.3)	
N 2	90 (23)	31 (13)	
N 3	17 (4.3)	0 (0)	
Distant metastasis—M, *n* (%)			0.140
M 0	377 (96.2)	228 (98.3)	
M 1	15 (3.8)	4 (1.7)	
UICC Stage, *n* (%)			<0.001
UICC I	114 (29.1)	117 (49.2)	
UICC II	73 (18.6)	39 (16.4)	
UICC III	53 (13.5)	35 (14.7)	
UICC IVA	122 (31.1)	43 (18.1)	
UICC IVB	15 (3.8)	0 (0)	
UICC IVC	15 (3.8)	4 (1.7)	
Recurrence, *n* (%)			0.359
No recurrence	290 (74)	166 (69.7)	
Recurrence	102 (26)	72 (30.3)	
Secondary carcinoma, *n* (%)			0.745
No secondary carcinoma	346 (88.3)	208 (87.4)	
Secondary carcinoma	46 (11.7)	30 (12.6)	
Therapy group, *n* (%)			<0.001
Primary surgical	320 (81.8)	228 (95.8)	
Primary radio(chemo-)therapy	53 (13.6)	8 (3.4)	
Palliative	18 (4.6)	2 (0.8)	
TTI (days)			<0.001
Mean ± SD	20.1 ± 17	33.5 ± 22	

Significant differences were observed in the distribution of TNM classification and UICC staging between the two groups. In the non-MTB group, 137 (57.6%) patients were classified as T1, while in the MTB group, 138 (35.2%) patients had T1 carcinomas. Tumors classified as T2 were distributed evenly, with 63 (26.5%) in the non-MTB and 116 (29.6%) in the MTB group. T3 tumors were present in 15 (6.3%) and 52 (13.3%) in the two groups, while T4 tumors numbered 23 (9.7%) in the non-MTB group and 85 (21.7%) in the MTB group (*p* < 0.001). N status also differed significantly between the groups (*p* < 0.001). M status was evenly distributed with 228 (98.3%) patients having M0 in the non-MTB group and 377 (96.2%) patients in the MTB group (*p* = 0.140, Table 1).

This resulted in a total of 117(49.2%) UICC I staged patients in the non-MTB group and 113 (28.8%) in the MTB group (*p* *<* 0.001). There was no significant difference in UICC II and III staging between the two groups (both *p* > 0.05). UICC IVA and IVC comprised to 43 (18.1%) and 4 (1.7%) patients in the non-MTB group, while in the MTB group, 122 (31.1%) patients had UICC IVA carcinoma, 15 (3.8%) had UICC IVB carcinoma, and 15 (3.8%) had UICC IVC carcinoma. This resulted in a significantly higher proportion of UICC IV-staged patients in the MTB group (*p* *<* 0.001, Table 1).

### 3.2 Survival analysis of the cohort

3-YSR was 73.1% in the non-MTB group and 67.1% in the MTB group. This difference was not statistically significant (*p* *=* 0.112). 3-YDFSR was 73.8% in the non-MTB group and 76.5% in the MTB group, again without significant difference (*p* *=* 0.447).

**Figure 1:**
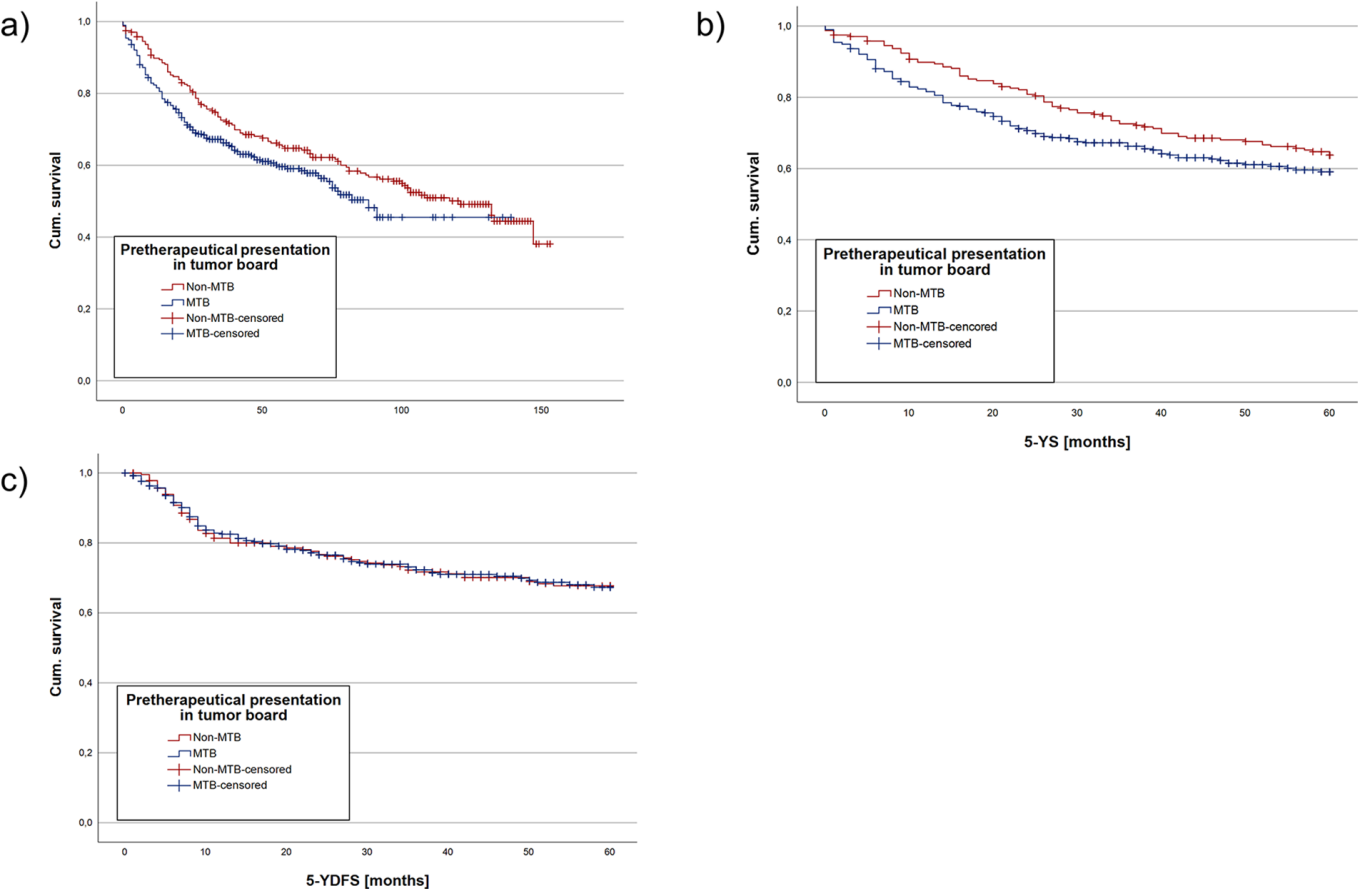
Kaplan meier analysis regarding the whole cohort divided into a group with pretherapeutic MTB presentation and without pretherapeutic MTB presentation. **(a)** showing the OS, **(b)** the mean 5-YS and **(c)** the mean 5-YDFS. NS, not significant, * = *p* < 0.05, ** = *p* < 0.01, *** = *p* < 0.001.

Median follow-up comprised for 65 months (CI: 59.6; 70.4) in the cohort. Median follow-up in the non-MTB group was 107 months (CI: 100.5; 133.5) and 52 months (CI: 48.3; 55.7) in the MTB group. Kaplan-Meier analysis of the study population showed a mean OS of 96.4 months (CI: 88.6; 104.2) in the non-MTB group, in comparison to 81.7 months (CI: 74.6; 88.6) in the MTB group. According to log-rank testing, the non-MTB group exhibited a significantly higher mean OS (*p* *=* 0.046) (Figure 1a).

Estimated mean 5-YS was analyzed due to the differing mean follow-up times of the two groups, with longer follow-up in the non-MTB group. Kaplan-Meier analysis for 5-YS showed no statistically significant difference between the non-MTB (*p* *=* 0.086, Figure 1b). Cox regression model yielded no significant difference between both groups regarding pretherapeutic MTB presentation as well as TTI (both *p* *>* 0.05). However, age at initial diagnosis (HR 1.03, *p* *<* 0.001, Table 2) and UICC stage did make a statistically significant difference (*p* *<* 0.001).

**Table 2:** Kaplan-Maier analysis and Cox regression of 5-YS data. The data for 5-YS were compared in the MTB and non-MTB group. First estimated median 5-YS was analyzed, followed by a multivariate Cox regression.

Parameter	*N*	in%	Estimated median 5-YS (months)	95% CI range (months)	Log rank *p* value
Overall cohort					
MTB	392	62.23	47.2	44.7–49.7	0.086
Non-MTB	238	37.78	43	40.7–45.3	
UICC I					0.714
MTB	114	49.35	53.4	50.7–56.1	
Non-MTB	117	50.65	53.4	50.7–56.1	
UICC II					0.554
MTB	73	65.18	48	43.2–52.8	
Non-MTB	39	34.82	47.1	41.2–53	
UICC III					0.258
MTB	53	60.23	46.4	40.7–48.2	
Non-MTB	35	39.78	40.7	33.2–48.2	
UICC IV					0.202
MTB	122	26.06	39.2	32.4–46	
Non-MTB	43	73.94	32.8	28.4–37.2	
Parameter			Multivariate Cox regression HR	95% CI	*p* value
MTB presentation			0.81	0.6–1.09	0.164
Age			1.03	1.02–1.04	<0.001
TTI			0.998	0.99–1.01	0.578

Kaplan-Maier analysis of mean 5-YDFS detected no significant difference between the two groups (*p* *=* 0.995, Figure 1c). Cox regression model resulted in no significant difference in 5-YDFS due to pretherapeutic presentation in MTB, age at initial diagnosis and TTI (all *p* *>* 0.05, Table 3). However, UICC stages had a statistically significant impact on 5-YDFS in Cox regression model (*p* *=* 0.019).

**Table 3:** Kaplan-Maier analysis and Cox regression of 5-YDFS data. The data for 5-YDFS were compared in the MTB and non-MTB group. First estimated median 5-YS was analyzed, followed by a multivariate Cox regression.

Parameter	*N*	in%	Estimated median 5-YDFS (months)	95% CI range (months)	Log rank *p* value
Overall cohort					
MTB	392	62.23	46.2	43.3–49	0.995
Non-MTB	238	37.78	46.3	44–48.7	
UICC I					0.706
MTB	114	49.35	49.2	45.6–52.8	
Non-MTB	117	50.65	50.5	47–53.9	
UICC II					0.93
MTB	73	65.18	49.3	44.5–54.1	
Non-MTB	39	34.82	48.7	42.1–55.3	
UICC III					0.371
MTB	53	60.23	46.7	40.7–52.7	
Non-MTB	35	39.78	41.9	33.7–50.2	
UICC IV					0.963
MTB	122	26.06	39.9	35–48	
Non-MTB	43	73.94	40.3	32.6–48	
Parameter			Multivariate Cox regression HR	95% CI	*p* value
MTB presentation			0.93	0.67–1.3	0.68
Age			1	0.99–1.01	0.715
TTI			0.99	0.98–1	0.059

### 3.3 Survival analysis of the UICC stages

A survival analysis was conducted among the UICC stages due to the differing distribution between the MTB and non-MTB groups.

**Figure 2:**
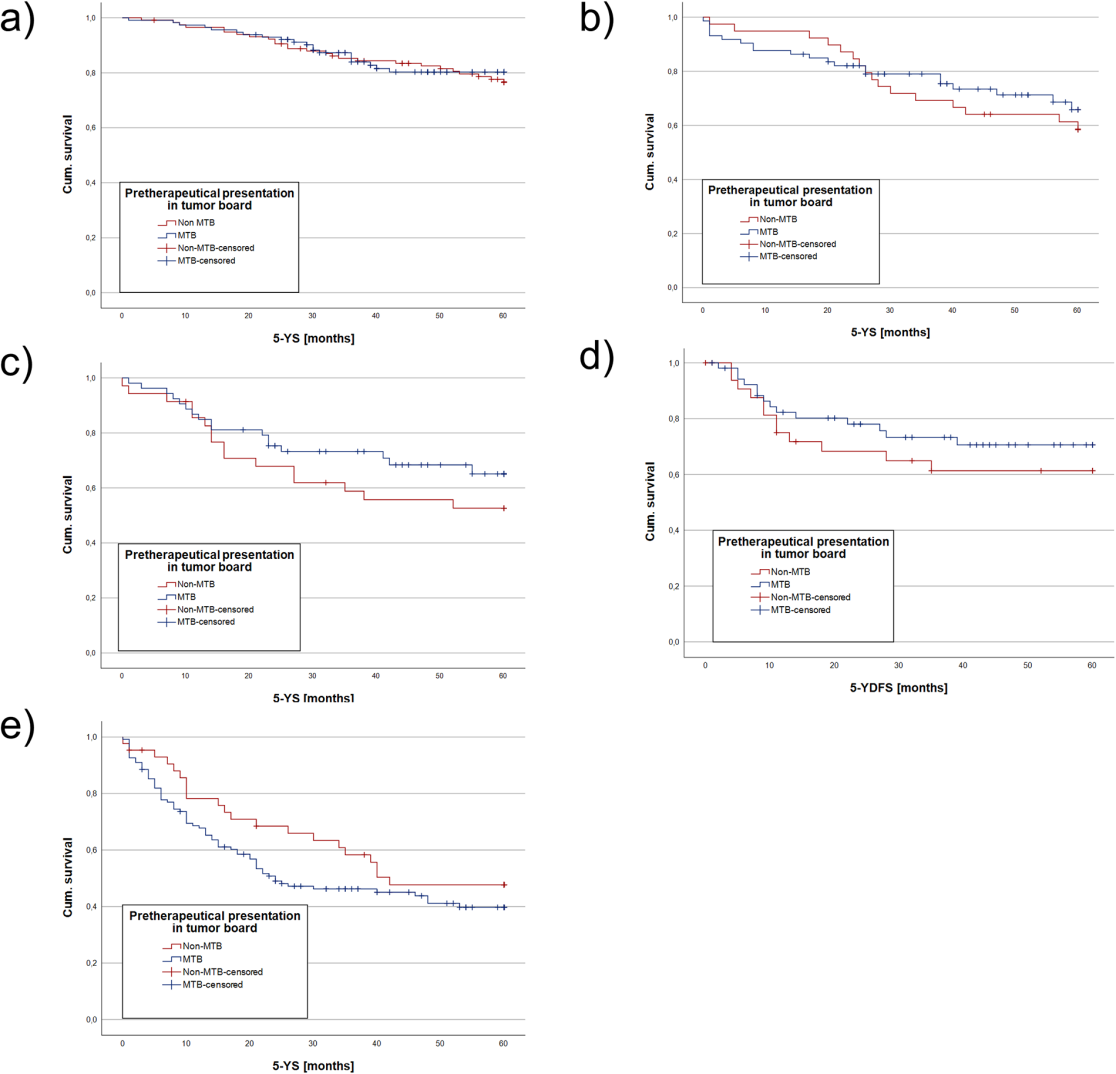
Kaplan Meier analysis of estimated mean 5-YS and 5-YDFS, comparing the MTB and non-MTB groups. Depicted are Kaplan Meier analysis for UICC I mean 5-YS **(a)**, for UICC II mean 5-YS **(b)**, for UICC III mean 5-YS **(c)** and 5-YDFS **(d)**, for UICC IV mean 5-YS **(e)**. NS, not significant, * = *p* < 0.05, ** = *p* < 0.01, *** = *p* < 0.001.

For UICC I, 117 non-MTB and 114 MTB patients were analyzed. 3-YSR was 85.5% (non-MTB) vs. 85.1% (MTB) (*p* *=* 0.935) and 3-YDFSR was 78.6% (non-MTB) vs. 80.7% (MTB) (*p* *=* 0.696). Kaplan-Meier analysis showed a mean 5-YS of 53.4 months for both groups (*p* *=* 0.714 Table 2 and Figure 2a) and a mean 5-YDFS of 49.2 (non-MTB) vs. 50.5 months (MTB) (*p* *=* 0.706, Table 3).

There were 39 non-MTB and 73 MTB patients included in UICC II stage. 3-YSR was 69.2% in the non-MTB and 79.5% in the MTB group (*p* *=* 0.228). 3-YDFSR accounted for 79.5% (non-MTB) vs. 82.2% (MTB) (*p* *=* 0.727). The estimated mean 5-YS showed 47.1 months for both groups (*p* *=* 0.554 Table 2 and Figure 2b), whereas the mean 5-YDFS was 48.7 (non-MTB) and 49.3 months (MTB) (*p* *=* 0.93, Table 3).

UICC III staged patients accounted for 35 non-MTB and 53 MTB patients. 3-YSR was 60% (non-MTB) vs. 73.6% (MTB) (*p* *=* 0.181) and 3-YDFSR was 64.7% (non-MTB) vs. 75.5% (MTB) (*p* = 0.279). Kaplan-Meier analysis yielded a mean 5-YS of 40.7 (non-MTB) vs. 46.4 months (MTB) (*p* = 0.258, Table 2 and Figure 2c), the mean 5-YDFS was 41.9 (non-MTB) vs. 46.7 months (MTB) (*p* = 0.371, Table 3 and Figure 2d).

For UICC IV, 43 non-MTB and 122 MTB patients were analyzed. The 3-YSR was 55.3% in the non-MTB and 45.4% in the MTB group (*p* *=* 0.234). The 3-YDFSR was 63.8% (non-MTB) vs. 71.1% (MTB) (*p* *=* 0.348). Kaplan-Meier analysis accounted for a mean 5-YS of 39.2 in the non-MTB and 32.8 months in the MTB group (*p* = 0.202, Table 2 and Figure 2e), and the mean 5-YDFS was 40.3 (non-MTB) vs. 39.9 months (MTB) (*p* *=* 0.963, Table 3).

### 3.4 Time to treatment initiation

**Figure 3:**
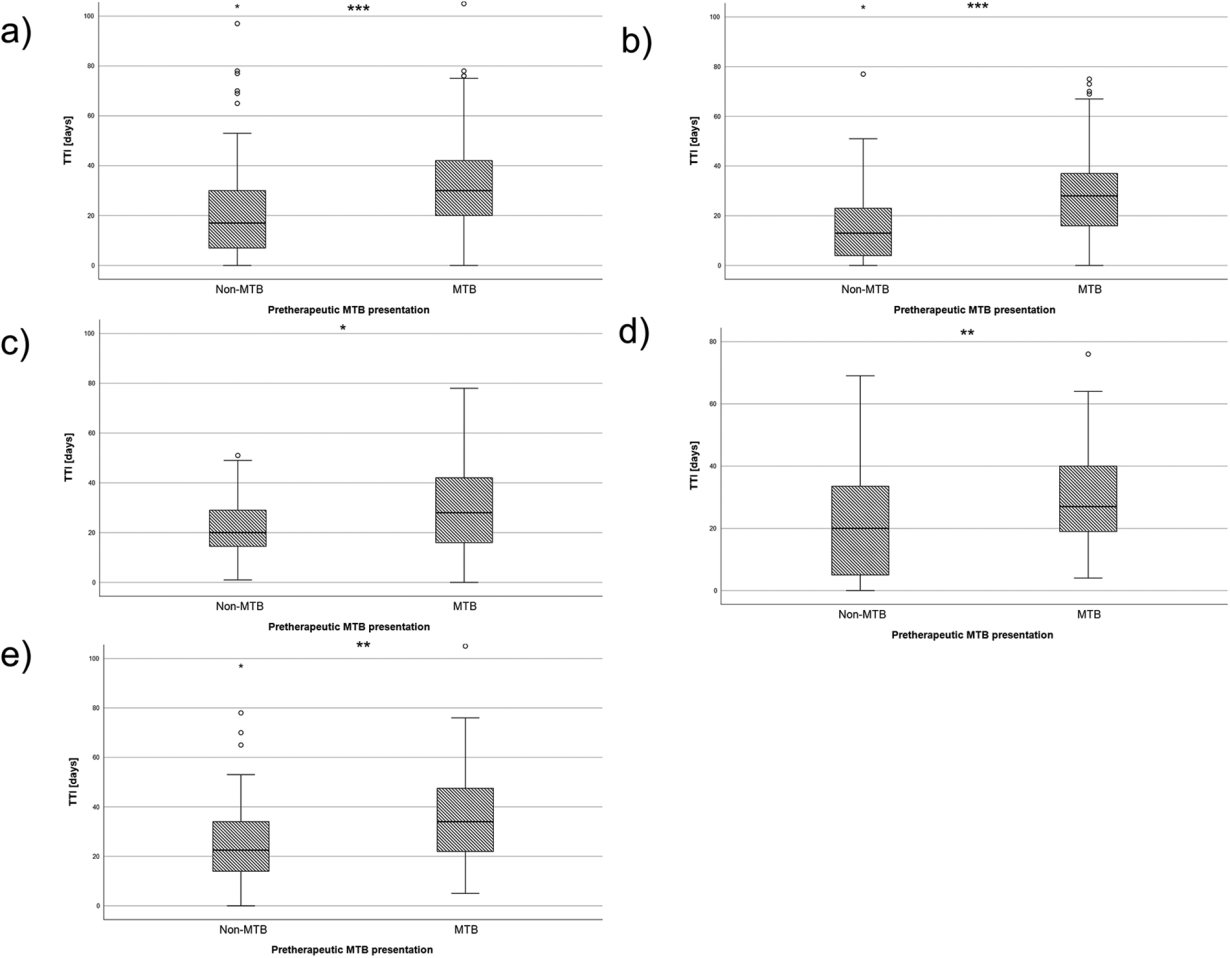
Box plots depicting the TTI for the two groups of MTB and non-MTB presentation. **(a)** shows the TTI of the whole cohort, **(b)** shows the TTI of all UICC I patients, **(c)** shows the TTI of all UICC II patients, **(d)** shows the TTI of all UICC III patients and **(e)** shows the TTI of all UICC IV patients. NS, not significant, * = *p* < 0.05, ** = *p* < 0.01, *** = *p* < 0.001.

As a preliminary measure, the time to MTB presentation (TMTB) was determined for each therapy group. The mean TMTB was 15.7 days for the surgically treated group, 14.2 days for the radio(chemo)therapy-treated group and 35.6 days for the palliative group. Subsequently, mean TTI was calculated for the cohort. Patients undergoing surgery as first-line therapy had a TTI of 25.9 days (CI: 24.3; 27.5), while patents treated with a radio(chemo)therapy had a TTI of 46 days (CI: 40.4; 51.5) and palliative patients had a mean TTI of 50.4 days (CI: 24.3; 76.6). The effect of MTB presentation was evaluated next. Patients without MTB presentation had a mean TTI of 20.1 days (CI: 17.9; 22.4), whereas the group with pretherapeutic presentation in MTB had a mean of 33.5 days (CI: 31.3; 35.7, *p* *<* 0.001, Figure 3a and Table 1).

A similar pattern was observed when the cohort was divided into UICC stages. In UICC I stage, the TTI was 16.4 days was significantly shorter in the non-MTB group (CI: 13.4; 19.4) and30 days in the MTB group (CI: 26; 34) (*p* *<* 0.001, Figure 3b). For UICC II staged patients, the TTI was 22.4 days (CI: 18.5; 26.4) in the non-MTB group and 31.7 days (CI: 26.4; 37) in the MTB group (*p* *=* 0.02, Figure 3c). Measurement in UICC III stage yielded 20.7 days (CI: 15; 26.3) in the non-MTB group and 30.6 days (CI: 26.1; 35) in the MTB group, which showed a statistically significant difference between both groups (*p* *=* 0.006, Figure 3d). The same results were found for UICC IV staged patients, with a TTI of 27.1 days (CI: 20.1; 34) in the non-MTB group and 38.1 days (CI: 33.8; 42.3) in the MTB group. This yielded a statistically significant difference (*p* = 0.009, Figure 3e).

### 3.5 Adherence with national guideline

The national guideline for the management of oral cavity carcinoma was published in December 2012. Consequently, our analysis focused on patients treated after this date to evaluate their adherence to the guideline. A total of 487 patients were included in the analysis, 114 did not receive treatment in accordance with the national guideline, while 373 patients were treated following the guideline.

**Figure 4:**
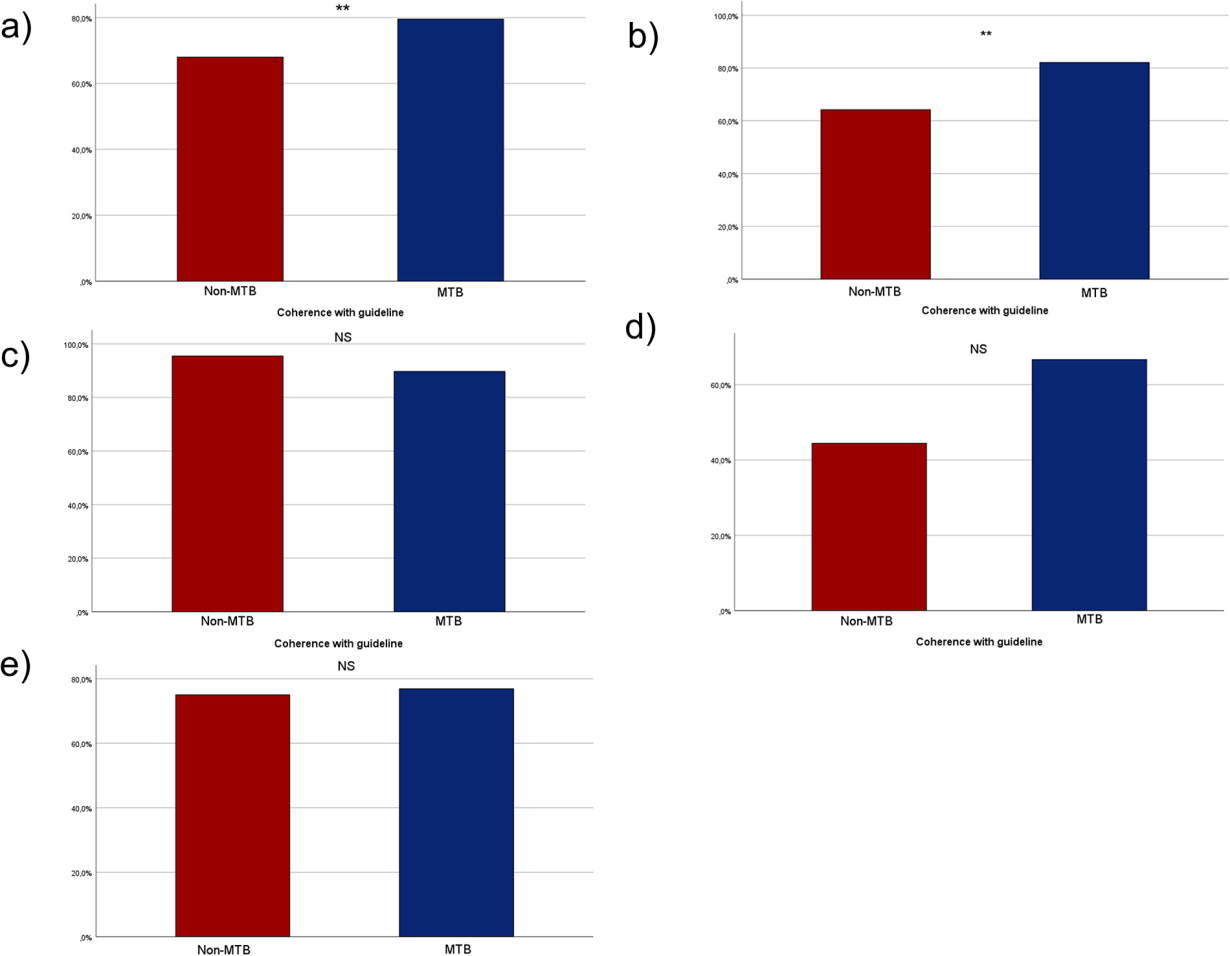
Bar charts depicting the adherence to national guideline in the two groups of MTB and non-MTB presentation. **(a)** shows the adherence in the whole cohort, **(b)** shows the adherence with the national guideline among all UICC I patients, **(c)** shows the adherence of all UICC II patients, **(d)** shows the adherence of all UICC III patients and **(e)** shows the adherence of all UICC IV patients. NS, not significant, * = *p* < 0.05, ** = *p* < 0.01, *** = *p* < 0.001.

In the non-MTB group, treatment of 40 patients (32%) did not adhere to the guideline, while 85 patients (68%) received treatment in accordance with the guideline. In the MTB group 74 patients (20.4%) were not and 288 patients (79.6%) were treated following the national guideline. Notably, statistically significant more patients cohered with the guideline when presented to the MTB prior to the initiation of therapy (*p* = 0.009, Figure 4a).

Of the patients treated primarily surgically, 180 (78.9%) in the non-MTB group underwent neck dissection, whereas 286 (90.5%) in the MTB group underwent neck dissection. This results in a significant difference with *p* < 0.001.

In total, 71 patients did not comply with the MTB recommendation. Among them, 23 patients (32.4%) did not adhere to the MTB recommendations at their own request and for *n* = 32 patients (45.1%), no documented reasons were available. Additionally, nine patients (12.8%) were not treated following the MTB resolution due to changes in TNM staging after presentation without reevaluation in the MTB before treatment. Finally, seven patients (9.9%) passed away before the treatment was initiated.

For UICC I stage 43 patients (64.2%) in the non-MTB group and 92 patients (82.1%) in the MTB group cohered with the guidelines, demonstrating statistically significant higher compliance with the guideline in the MTB group (*p* < 0.007, Figure 4b). In UICC II stage, 21 patients (95.5%) in the non-MTB group and 61 patients (89.7%) in the MTB group were treated in accordance with the guideline (*p* = 0.41, Figure 4c). In UICC III stage, eight patients (44.4%) in the non-MTB group cohered with the guideline, compared to 34 patients (66.7%) in the MTB group (*p* = 0.097, Figure 4d). Among UICC IV staged patients, 12 patients (75%) in the non-MTB group and 80 patients (76.9%) in the MTB group received treatment in accordance with the guideline (*p* = 0.866, Figure 4e).

## 4 Discussion

This study is the first to evaluate the impact of pretherapeutic MTB presentation on OS, DFS, mean 5-YS, and mean 5-YDFS in oral cavity cancer patients. MTBs are crucial in cancer treatment within specialized tumor centers, but evidence regarding their impact on survival and guideline adherence, especially in head and neck tumors, is limited.

The study focused solely on oral cavity cancer, distinguishing it from other head and neck cancers such as oropharyngeal, hypopharyngeal, laryngeal or salivary gland cancers (19, 21). This differentiation is crucial due to varying tumor characteristics and disease courses. This is reflected in different survival times and differences in the TNM classification at the time of initial diagnosis (2). It is noteworthy that the study demonstrated no survival advantage for the cohort of patients who were presented to the tumor board prior to therapy. However, the TTI was significantly prolonged, despite the therapy being more guideline-compliant with the presentation in the MTB.

The cohort in this study included 630 patients with primary oral cavity cancer diagnoses, unlike previous studies that often included mixed head and neck cancer cases. Liu et al. included 224 head and neck squamous cell carcinoma (HNSCC patients) (14) and Rangabashyam et al. included 221 patients with HNSCC (22). Larger multicenter studies by Meltzer et al. (*n* = 3,081) (21) and Hansen et al. (*n* = 28,293) covered all head and neck cancer sites (23). The largest studies examining the impact of MTB approaches in oral cavity cancer were conducted in Taiwan from Liao et al. 2016 including 1,616 patients between 1996 and 2011 (24), and Tsai et al., which was a nation-wide study in Taiwan with nearly 17,000 patients (25).

This study employed a retrospective design, as was the case with many previous studies comparing patients treated pre- and post-implementation of MTBs (21, 23, 24). The groups of non-MTB and MTB presentation were predetermined due to the implementation of an MTB at the University Medical Center Freiburg in 2014. To our knowledge, only one prospective study by Wheless et al. exists, which examined changes in cancer classification and treatment regimen over three months. In that study, 66% of patients had no change in diagnosis or treatment, while 27% had changes due to MTB presentation, with stronger effects in malignant tumors. However, it did not compare treatment to local guidelines (26). Unfortunately, this study did not compare the treatment to local guidelines.

Tsai et al. conducted a nationwide retrospective study on oral cavity cancer, postulating a beneficial impact of multidisciplinary treatment (MDT) on OS in nearly 17,000 patients from 2004 to 2010 (25). The study provides a comprehensive overview of oral cavity cancer patients and their outcomes in Taiwan. Using the 6th UICC classification, they found no significant improvement in OS for stages I-III, but a notable improvement for stage IV disease. This might indicate that center-based cancer treatment and MDT and center-based treatment are particularly beneficial for advanced stages. Compared to Tsai et al., our study had a lower proportion of UICC IV patients (51.6% vs. 31.6%). Additionally, Tsai et al. focused on MDT rather than MTB, limiting direct comparison with our study.

Friedland et al. similarly reported no significant improvement in 5-YSR for stages I-III but a better 5-YSR for stage IV among 726 primary head and neck cancer patients (1996–2008) (19). Neither study evaluated the DFS. Our results align with Tsai et al. for stages I-III, showing no significant improvement in 3-YSR, 3-YDFSR, mean 5-YS, or 5-YDFS. In UICC III stage, our MTB group showed a trend towards higher 3-YSR and 3-YDFSR, but without statistical significance. Unlike Tsai et al., we did not observe improved outcomes in UICC IV patients and multivariate analysis showed no impact of MTB presentation on mean 5-YS and 5-YDFS across all stages.

Liao et al. (2015) reported a higher 5-YSR for oral cavity cancer patients in the MTB group, focusing on those undergoing primary surgical treatment (24). This differs from our study, where all patients were included, as in other previously mentioned studies (19, 23, 25). Liao et al. discussed the higher rate of neck dissections in the MTB group and higher rates of adjuvant radiotherapies in the MTB-group as possible factors for a better 5-YSR (24). Our study also showed a higher rate of neck dissections performed in patients treated primarily with surgery (90.5% in the MTB vs. 78.9% in the non-MTB group, *p* < 0.001). It has been demonstrated that neck dissection is a pivotal aspect of oral cavity cancer treatment (27). This is crucial in comparing the data of Liao et al. with our study. Mandatory recommendations for neck dissections in Germany were implemented with the guideline in 2012, which resulted in most surgically treated patients undergoing neck dissection. This might explain the difference of neck dissection rates between both groups in our study.

In 2021, Meltzer and colleagues published a retrospective study encompassing all head and neck cancer sites. The study demonstrated a tendency towards improved 3-YSR and 3-YDFSR outcomes for all stages, although statistical significance was not reached (21). Our data aligned with this tendency in UICC stages II and III concerning 3-YSR (69.2% in the non-MTB group vs. 79.5% in the MTB group). The survival benefits from other retrospective studies could not be confirmed in our study for various reasons. Due to the different cohorts (all HNSCC patients included or older cohorts in which neck dissections were not standard), the partly different results can be explained.

The retrospective study by Hansen et al. (2020) indicated that the MTB-associated approach delayed treatment initiation by 18 days (23). Our study observed a similar delay of approximately 13 days across all UICC stages in the MTB group compared to the non-MTB group, with the greatest delay in UICC I patients with over 13 days and UICC IV patients with 11 days. Metzler et al. reported a comparable TTI of 32 days in the non-MTB group vs. 33 days in the MTB group (21). Evidence suggests that TTI impacts HNSCC outcomes, as longer TTI can lead to tumor progression and clinical-to-pathological upstaging, associated with poorer survival (28, 29, 30, 31).

Rygalski et al., in a study of over 37,000 HNSCC patients, demonstrated that delayed surgery significantly affected OS, with the highest hazard ratio 67 days post-diagnosis and an incremental 4.7% increase for each additional 30-day delay (28). Notably, the data published by Rygalski et al. were not specific to oral cavity carcinoma. However, the mean delay in our study was considerably shorter than 67 days in both groups, namely those with and without MTB presentation. Taking this in consideration, the delay of approximately 13 days may not affect the OS or DFS.

A postulated goal of the MTB is to reduce TTI. This assumption was supported by a systematic review by Prades et al. in 2015 (12). Other beneficial side effects of the MTB, such as improving interdisciplinary work, providing a supervisory body, educating younger colleagues, and enhancing the quality of life through the interdisciplinary approach, are challenging to measure and were not part of this study.

As a retrospective study, the design, data collection, and potential sampling bias are subject to several limitations. The study was conducted at a single center, which may have influenced the results due to the specific surgical and medical skills of the physicians at that clinic. Additionally, the introduction of MTB during observation may have resulted in a higher likelihood of the non-MTB group being treated before 2014, which could affect the measured OS and DFS in the two groups. Furthermore, we cannot rule out that differences arise between the groups due to a tendency towards treatment at different time points. Nevertheless, in comparison to registry studies on this subject, the tumor characteristics and treatments of the patients could be more comprehensively elucidated, given that all data were collected from a single center. Furthermore, in addition to survival and DFS, other factors, such as TTI and adherence to the national guideline, were also examined.

Nevertheless, our study found that the MTB presentation before treatment initiation led to a higher level of coherence to the national guideline. This improvement was mainly evident in the group of UICC I patients, where 82.1% of the patients in the MTB group were treated according to the guideline, compared to 64.2% in the non-MTB group. This primarily involved resection with neck dissection, which was more frequently omitted in the non-MTB group. This finding is consistent with the results previously reported by Liao and colleagues (24).

## 5 Conclusion

In conclusion, this study has revealed both positive and negative effects of MTB presentation in patients with oral cavity cancer. The findings indicated a delay in treatment initiation in the MTB group, while patients were more frequently treated in accordance with the national guideline this group. This did not result in a difference in 3-YSR, 3-YDFSR, mean 5-YS, or mean 5-YDFS. The observed delay in treatment initiation may be offset by the improved coherence with the guideline. Other potential beneficial effects of MTB's, such as acting as a control body, improving interdisciplinary work and providing educational opportunities, were not measured in this study. The impact of better OS due to a center-based treatment could not be measured due to the single-center nature of the study. Further multicenter studies may help address questions about the effects of TTI and guideline coherence related to MTB presentation on survival of oral cavity cancer patients.

## Data Availability

The datasets presented in this study can be found in online repositories. The names of the repository/repositories and accession number(s) can be found in the article/Supplementary Material.
